# Flexible Bond Wire Capacitive Strain Sensor for Vehicle Tyres

**DOI:** 10.3390/s16060929

**Published:** 2016-06-21

**Authors:** Siyang Cao, Simon Pyatt, Carl J. Anthony, Ammar I. Kubba, Ali E. Kubba, Oluremi Olatunbosun

**Affiliations:** 1 Department of Mechanical Engineering, University of Birmingham, Birmingham B15 2TT, UK; siyangfox@gmail.com (S.C.); aik368@bham.ac.uk (A.I.K.); o.a.olatunbosun@bham.ac.uk (O.O.); 2 School of Physics and Astronomy, University of Birmingham, Birmingham B15 2TT, UK; s.pyatt@bham.ac.uk; 3 Fusion Innovations Ltd., Research and Innovation Services, Birmingham Research Park, Vincent Drive, Edgbaston, Birmingham, B15 2SQ, UK; A.E.S.Kubba@bham.ac.uk

**Keywords:** strain sensor, flexible, wire bonding, PDMS

## Abstract

The safety of the driving experience and manoeuvrability of a vehicle can be improved by detecting the strain in tyres. To measure strain accurately in rubber, the strain sensor needs to be flexible so that it does not deform the medium that it is measuring. In this work, a novel flexible bond wire capacitive strain sensor for measuring the strain in tyres is developed, fabricated and calibrated. An array of 25 micron diameter wire bonds in an approximately 8 mm × 8 mm area is built to create an interdigitated structure, which consists of 50 wire loops resulting in 49 capacitor pairs in parallel. Laser machining was used to pattern copper on a flexible printed circuit board PCB to make the bond pads for the wire attachment. The wire array was finally packaged and embedded in polydimethylsiloxane (PDMS), which acts as the structural material that is strained. The capacitance of the device is in a linear like relationship with respect to the strain, which can measure the strain up to at least ±60,000 micro-strain (±6%) with a resolution of ~132 micro-strain (0.013%). In-tyre testing under static loading has shown the ability of the sensor to measure large tyre strains. The technology used for sensor fabrication lends itself to mass production and so the design is considered to be consistent with low cost commercialisable strain sensing technology.

## 1. Introduction

The concept of the “intelligent tyre” has emerged from the beginning of this century to meet the demands of a safe and comfortable driving experience, as well as the requirement of reduced emission from the vehicle; hence, the tyre industry and tyre research has focused more and more on the physical quantities that can be measured by the “intelligent tyre”. Among these physical quantities that the “intelligent tyre” detects, the friction force between the surfaces of the tyre and road is one of the most significant physical quantities for the tyre behaviour and plays an important role for vehicle simulation, manoeuvrability of the vehicle and safety of the driving experience [1,2]. Several models for calculating and analysing the friction have been developed, such as slip/force model, lumped model and distributed model [3]. Meanwhile, finite element analysis (FEA) software has also been employed for simulating the friction based on these models by many researchers [4,5,6]. However, the friction between the surfaces of tyre and road are affected by many factors, such as the environmental characteristics, the inflation of the tyre, and the load of the tyre, and are difficult to measure directly [1].

Alternatively, the strain of the tyre has become another possible approach to investigate the frictions between the surfaces of the tyre and road [6,7]. Initially, foiled strain sensors were employed for measuring the strain in the tyres [6]. However, compared with the tyre rubber, the foiled strain sensor has higher stiffness, so it will inevitably have a significant effect on the strain behaviour of the sensing surface of the tyre; making measuring the strain accurately and reliably difficult [8,9].

There have been several attempts reported in the literature to develop strain sensing technology to overcome the issues associated with the high stiffness of commercial strain sensors. Alexander and Rajamani developed a novel PVDF-based piezoelectric sensor which can measure the lateral deformation in tyres [10]. Sergio *et al.* [11] and Matsuzaki *et al.* [12] adopted the steel wires in the tyre itself as a part of sensing element. When the tyre is deflected, the distance between the sensing wires and the length of the sensing wires will be compressed or expanded, causing a change in the capacitance and resistance. By measuring the variations, the strain in the sensing area can be calculated. Matsuzki *et al.* [8] also developed a rubber based capacitive sensor to reduce the influence that is brought about by the metallic structure of the sensor. Tuononen [13] employed a position sensitive detector (PSD) to measure the deformation in the tyre carcass. Based on the calculation of the position of the light spot on the tread when the tyre deflected, the deformation of the tyre will be known.

However, the fabrication process and the installation of the previous strain sensing techniques are either too complicated for tyres or not capable of mass production, and there has been little or no development for these strain sensors in recent years. The stiffness of the sensor is still one of the main problems in tyre strain sensing: a fully flexible and complaint sensor is extremely important for measuring the strain in tyres precisely and is still desired to be developed. This paper will present a novel capacitive strain sensor, based for the first time on bond wire technology, that has sufficiently low stiffness compared with the tyre rubber that it can measure the strain in the tyre stably and reliably without any influence and disturbance to the sensing area. The fabrication process of the strain sensor aims to use commercially available processes that will result in reasonable cost and will allow the sensor to be introduced to the industry for mass production.

## 2. Design and Calculation of the Wire Bond Capacitive Strain Sensor

### 2.1. Sensor Design

Interdigital structuring and polydimethysiloxane (PDMS) embedding are the two main design concepts of the new sensor. An interdigital structure is widely used in capacitive sensors in different research areas [8,14,15] and can fully utilize the square space to generate a reasonable capacitance; meanwhile, the multiple fingers split the whole electrode plates in the original parallel capacitive structure into many small ones, which can increase the flexibility of the capacitor dramatically and adapt the sensor to the stiffness of the tyre, thus minimizing the influence to the sensing area in the tyre. PDMS is introduced and concerned to be employed as an embedding material for the whole sensor structure. The Young’s modulus of PDMS in different mixing ratios varies from approximately 360 KPa to 870 KPa, which is very flexible compared with the tyre rubber (typically 5–10 MPa [16]). Thus, it is believed that the PDMS as an embedding agent can efficiently maintain the flexibility of the sensor and protect the structure from potential damages.

**Figure 1:**
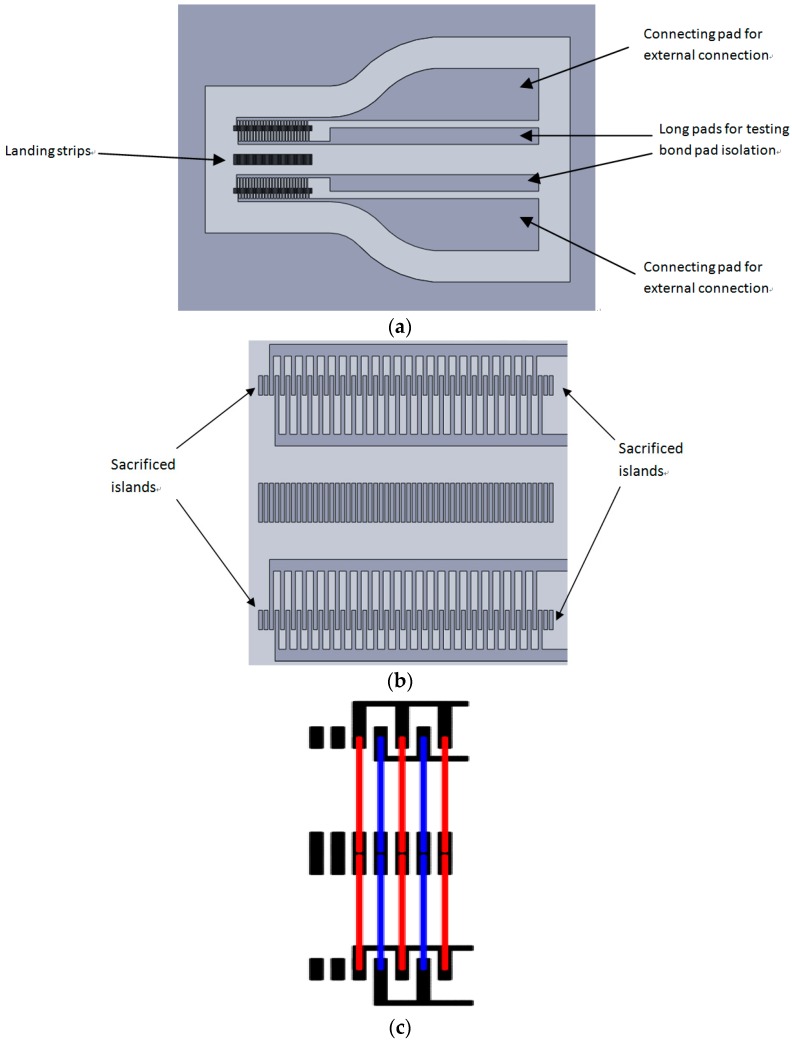
The bond wire sensor design: (**a**) layout of the metallic substrate for wire bonding; (**b**) the zoomed in picture of wire bonding area; and (**c**) the schematic bond wire configuration, red wires create one electrode and blue wires create the other.

Following the concept of the interdigital capacitive structure, a novel design was carried out. The capacitance is generated by multiple parallel wires, in which not only the number of fingers in the traditional interdigital capacitive structure can be increased, but the width of fingers is also reduced to a very small size. This controls the size of the sensor in a value that can measure the strain situation along the contact patch and in the same time minimize the stiffness of the structure. The wire “fingers” in the structure are an array of wires produced by a wire bonding process normally used for the electrical connection of microchips to their package [17]. The bond wires are connected to bond pads created on a flexible PCB. Figure 1a shows the design of the metallic bond pads created on a flexible PCB for landing the wire bonding feet. There are two wire arrays combined together by an array of landing strips and the bonding length in each wire array is 3 mm. All the gaps in the substrate are 40 micron and the width of the finger is 100 micron. Therefore, the spacing in the wire array is 140 micron. The landing islands at top and bottom are connected out for testing the isolation and the width of two electrodes are increased gradually from 300 micron to 5 mm by smooth curves for ease of connecting the external circuits. The central landing islands are used to create a double array of shorter bond wires rather than having long bond wires that span the whole sensor, as they are prone to drooping in the embedding process.

Additionally, there are two more sacrificed landing islands designed at each end of the wire bonding area, as shown in Figure 1b. The sacrificed islands will not contribute any capacitance to the design, but can keep dimensions of the gaps and fingers in the structure consistent during the fabrication process, especially for the fingers and islands at two ends of the design. Moreover, the sacrificed islands can provide some testing areas for the wire bonding process. Figure 1c shows a schematic configuration of the first five bond wires on the PCB.

### 2.2. Calculation

**Figure 2:**
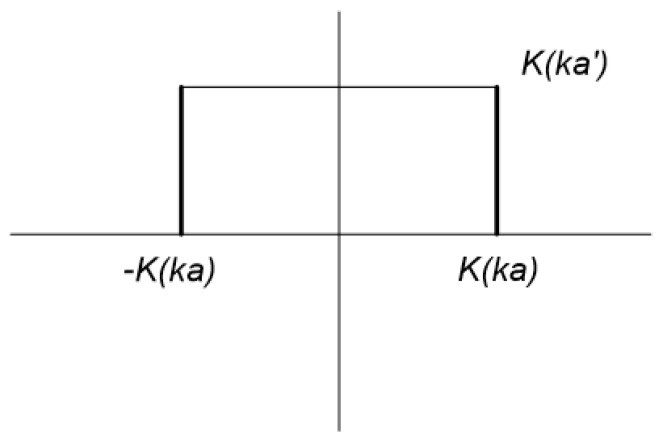
The rectangle structure that transferred from the non-uniform electric field based on Christoffel-Schwarz transformation [18].

In the bond wire capacitance design, there are totally 50 wires with 140 micron spacing that create 49 pairs of capacitors in parallel. It is a challenge to estimate the capacitance of this structure, the wire array cannot be considered as a summation of multiple pairs of capacitor in a parallel connection due to the intensive arrangement. The structure is considered to be more equivalent with a coplanar configuration. In the estimation, the wires are replaced with thin fingers with the width that equals to the diameter of the wire. So, for either upper half or bottom half of one pair of electrodes in the structure, employing the conformal mapping based on the Christoffel-Schwarz transformation can transfer the non-uniform electric field into a rectangle structure, which is shown in Figure 2. Hence, the capacitance $C_{I}$ of either upper half or bottom side of one pair of coplanar structure can be given by [18]:

(1) $$C_{I}=\varepsilon_{0}\varepsilon_{PDMS}L\frac{K\left({k_{a}}^{\prime }\right)}{2K\left(k_{a}\right)}$$

where $\varepsilon_{PDMS}=2.75$ is the dielectric constant of PDMS, L is the length of the wire, $K\left(k_{a}\right)$ is the total elliptic integral of the first kind and the elliptic integral $k_{a}$ is given by:

(2) $$k_{a}=\frac{s-2r}{s+2r}$$

where s is the spacing in the wire array and r is the radius of the wire.

(3) $${k_{a}}^{\prime }=\sqrt{1-{k_{a}}^{2}}$$

(4) $$\frac{K\left({k_{a}}^{\prime }\right)}{K\left(k_{a}\right)}=\{\begin{array}{c} \pi^{-1}\ln\left[2\cdot \frac{1+{\left(1-{k_{a}}^{2}\right)}^{0.25}}{1-{\left(1-{k_{a}}^{2}\right)}^{0.25}}\right]\;when\;0<k_{a}\leq 0.7\\ \pi {\left[\text{ln}\left(2\cdot \frac{1+{k_{a}}^{0.5}}{1-{k_{a}}^{0.5}}\right)\right]}^{-1}\;when\;k_{a}\geq 0.7 \end{array}$$

Based on the work of Igreja [19], the capacitance of the wire array $C_{a}$ can begiven by:

(5) $$C_{a}=2\cdot \left[\left(N-3\right)C_{I}+2\frac{2C_{E}C_{I}}{C_{E}+2C_{I}}\right]$$

where *N* is the total number of the wires and $C_{E}$ is the capacitance that generated by outer electrode/wire relative to the ground, which can be given by:

(6) $$C_{E}=-\frac{\pi \varepsilon_{0}\varepsilon_{PDMS}L}{ln\left(\frac{s-\sqrt{s^{2}-4r^{2}}}{2r}\right)}$$

If the wire curve can be treated as an arc and the height of the curve is h, the radius of the arc R can be solved by:

(7) $$R^{2}={\left(\frac{l}{2}\right)}^{2}+{\left(R-h\right)}^{2}$$

Hence, R is given by:

(8) $$R=\frac{l^{2}+4h^{2}}{8h}$$

where l = 3 mm is the bonding length and h is the height of the wire bonding.

Thus, L is given by:

(9) $$L=4R\pi \sin^{-1}\frac{\frac{l}{2}}{R}=\frac{l^{2}+4h^{2}}{2h}\pi \sin^{-1}\frac{4hl}{l^{2}+4h^{2}}$$

Thus, the estimation of the capacitance of the wire bond structure using Equations (5) and (6) is 7.818 pF. The sensitivity of the senor can be estimated by varying the initial wire spacing(s) around the nominal 140 microns of the design. By doing this, it was found that the capacitance strain relationship is essentially linear at this point, and the sensitivity can be calculated as being 25.8 fF per 10,000 micro strain. What has to be mentioned is, when the number of the wires is big enough (more than a hundred), the capacitance that is generated by the outer electrode/wire can be neglected, then Equation (5) can be simplified as:

(10) $$C_{a}=2\cdot \left(N-3\right)C_{I}$$

## 3. Fabrication Process of the Wire Bond Capacitive Strain Sensor

A special flexible PCB called Pyralux AP flexible circuit material from DuPont is selected as the substrate material. The material is a double-sided, copper-clad laminate while the all-polyimide composite of polyimide film is bonded to copper surface [20]. The thickness of the copper layer is 35 microns while the thickness of the polyimide layer between the copper laminates is 2 mil, which is 50.8 microns. The polyimide film makes the material capable of being wire bonded and will not affect any process on the material during the fabrication. A hybrid wet etching and laser machining process was used to achieve the bond pad dimensions of the pattern in the design. A laser machining process was used to remove most of the copper material without damaging the polyimide in the flexible PCB this was followed by a wet etching process used to clean the residual copper that is re-deposited in the laser machining process and ensure that the gaps in the pattern were electrically isolated.

**Figure 3:**
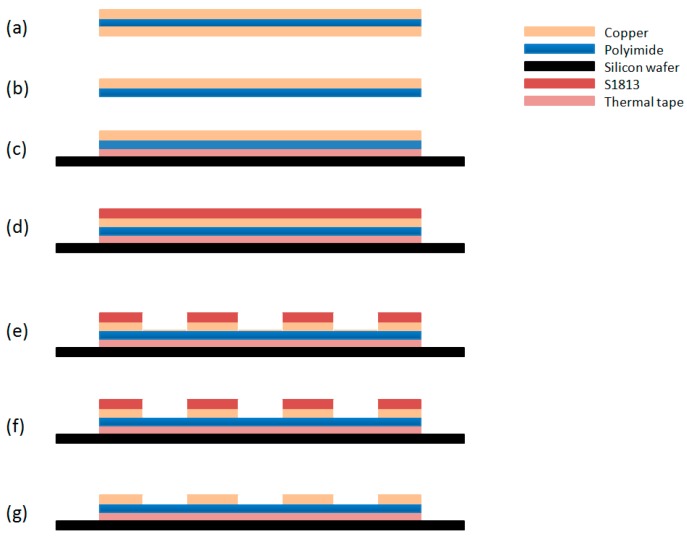
A schematic diagram of wet etching method: (**a**) the flexible PCB substrate; (**b**) etching down one side of the copper; (**c**) bond to silicon wafer; (**d**) coating and exposing a S1813 photoresist layer; (**e**) laser machining down most of copper layer; (**f**) cleaning off the rest of copper in the gaps; and (**g**) cleaning the photoresist layer.

As shown in Figure 3, one side of the copper layer of the flexible PCB was fully removed by etching in 10 wt % ferric chloride solution and then bonded on a silicon wafer using Nitto Denko Revalpha thermal release tape, which is shown in Figure 3b, c. The photoresist S1813 was poured on the wafer and spun at 500 rpm for 10 s, 2000 rpm for 30 s, 500 rpm for 10 s continuously and then baked on the hot plate at 80 °C for 30 min. As followed, the photoresist was fully exposed for 8 s, which is shown in Figure 3d. The photoresist was used to protect the copper surface that would be wire bonded from the laser machining debris. After being coated with photoresist S1813 and exposure, the flexible PCB was laser machined into the pattern that is shown in Figure 1aby a laser machine tool LS4 from LASEA, which is shown in Figure 3e. Then the PCB was etched in 10 wt % ferric chloride solution for 10 min to clean up the residual copper material for isolating all the gaps in the pattern, which is shown in Figure 3f. In the end, the photoresist was cleaned by soaking the sample in MF-319 for 15 min, as shown in Figure 3g.

**Figure 4:**
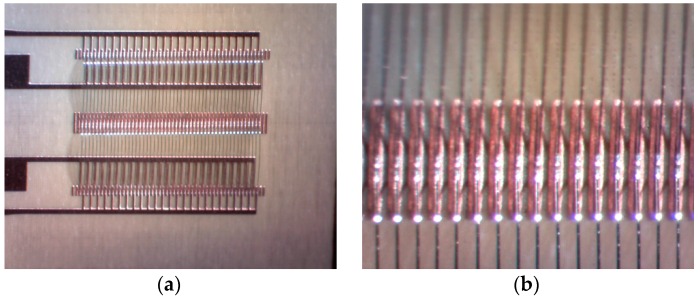
The configuration of wire bond structure: (**a**) before embedding in PDMS; and (**b**) the zoomed in bonding feet at land strips in the middle.

The wire bonding process was achieved using a bondjet BJ 820 automatic wedge bonder from HESSE Mechatronics (Paderborn, Germany). Following extensive trials for the best parallelism and stability of the bonding process, in the final design, the bond length was set to be 3mm and the bond height was set as 500 micron. Figure 4a shows the wire arrays before embedding in the PDMS and Figure 4b shows a zoomed in picture at the bonding islands landing strips in the middle of the design.

**Figure 5:**
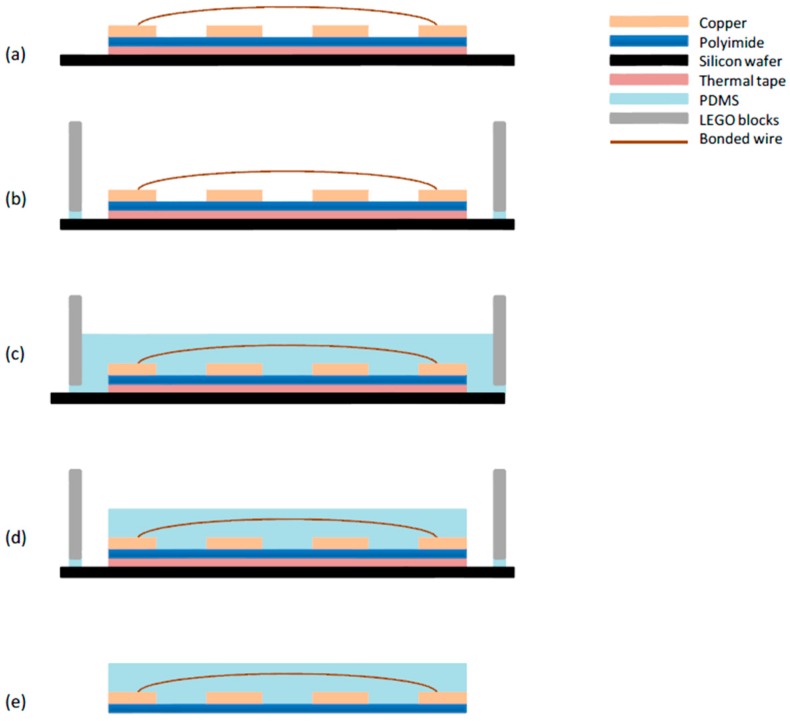
A schematic diagram of embedding process: (**a**) the copper pattern with wire bonded; (**b**) bonding the LEGO blocks on the silicon wafer by PDMS; (**c**) injecting PDMS into the mould and curing for 24 h; (**d**) cutting down the PDMS around sensor; and (**e**) baking at 160 °C to release the sensor from the silicon wafer.

The embedding process is the final step of the fabrication. A square frame with 24 mm inner edge was built by LEGO bricks and bonded to the silicon wafer by PDMS, which formed a mould that surrounded the wire bond structure still attached to the wafer by thermal tape, as shown in Figure 5b. Since the area is in a square shape and wire height is known, the volume of PDMS can be calculated for making a 0.5 mm thick PDMS layer that will cover the wire bonds, which was approximately 0.29mL. PDMS was injected by a syringe with 1mL capacity and 0.01mL precision into the mould (Figure 5c). There were some small bubbles produced because of mixing PDMS with the curing agent, so before injecting, the syringe was put upside down for an hour to release these bubbles. After that, the sample piece was left at room temperature for 24 h for curing. The LEGO bricks were then removed from the wafer and the PDMS around the wire bond structure was cut down to size, as shown in Figure 5d; the sample was then baked on a hot plate at 160 °C for 2 min to let the thermal tape release the sensor part from the wafer, as shown in Figure 5e.

**Figure 6:**
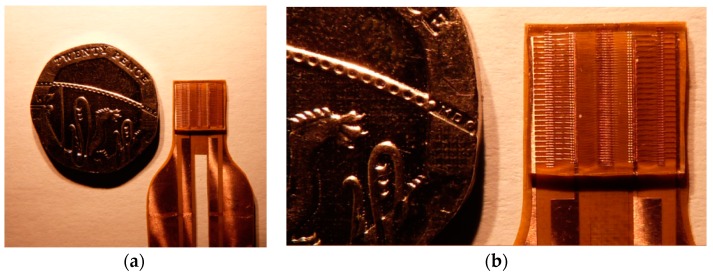
The fabricated final bond wire capacitor design: (**a**) compared with a 20 pence coin; and (**b**) the zoomed in image.

The fabricated final bond wire capacitor design after embedding in PDMS is shown in Figure 6.

## 4. Experiments and Calibration on the Wire Bond Capacitive Strain Sensor

### 4.1. Theory

**Figure 7:**
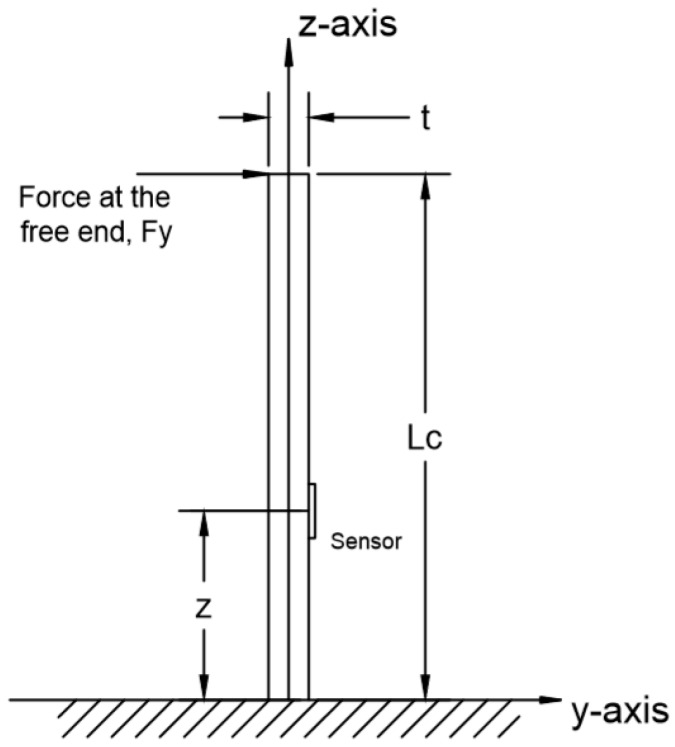
A schematic diagram for cantilever test for the strain sensor.

As a testing method that is employed in other research on strain sensors [21,22], a cantilever test is carried out for calibrating and testing the design whereby bending of the cantilever creates strain in the sensor attached near the base of the cantilever. To quantify the strain developed in the sensor during the test the configuration shown in Figure 7 is considered where the z-axis is taken along the direction of the cantilever, y-axis is perpendicular to the z-axis and parallel with the paper and the x-axis is vertical to the yz-plane, which is vertical to the paper. The sensor is bonded on one side of the cantilever at distance z from the fixed end. When a force $F=\;\left[\begin{array}{c} F_{x}\\ F_{y} \end{array}\right]$ is applied to the free end of the cantilever, the surfaces of both sides of the cantilever will either be in a compressed or stretched condition. From the Saint-Venant’s bending theory, the stress at the sensor $\sigma_{z}$ can be given by [23]:

(11) $$\sigma_{z}=-\left(\frac{F_{x}}{I_{y}}\cdot x+\frac{F_{y}}{I_{x}}\cdot y\right)\left(l_{c}-z\right)$$

where $F_{x}$ and $F_{y}$ are the forces in direction of x-axis and y-axis, $I_{x}$ and $I_{y}$ are the moments of inertia for the cantilever cross section for directions of x-axis and y-axis, while x and y stand for a point (x,y) at the cantilever cross section, respectively, and $l_{c}$ is the length of the cantilever.

If the force is only in the y-axis direction, Equation (11) can be simplified:

(12) $$\sigma_{z}=-\frac{F_{y}}{I_{x}}\cdot y\cdot \left(l_{c}-z\right)$$

thus, the strain at the sensor $\varepsilon_{z}$ is given by:

(13) $$\varepsilon_{z}=\frac{\sigma_{z}}{E_{c}}=-\frac{F_{y}}{E_{c}I_{x}}\cdot y\cdot \left(l_{c}-z\right)$$

Compared with the force, the deflection in the y-axis $D_{y}$ is easier to be measured in the experiment, while $D_{y}$ also has a relationship with the Young’s modulus of the cantilever $E_{c}$ and the area moment of inertia $I_{x}$ [24]:

(14) $$D_{y}=\frac{F_{y}l_{c}^{3}}{3E_{c}I_{x}}$$

then rearranging,

(15) $$\frac{F_{y}}{E_{c}I_{x}}=\frac{3D_{y}}{l_{c}^{3}}$$

and substituting into Equation (13), gives

(16) $$\varepsilon_{z}=-\frac{3D_{y}}{l_{c}^{3}}\cdot y\cdot \left(l_{c}-z\right)$$

**Figure 8:**
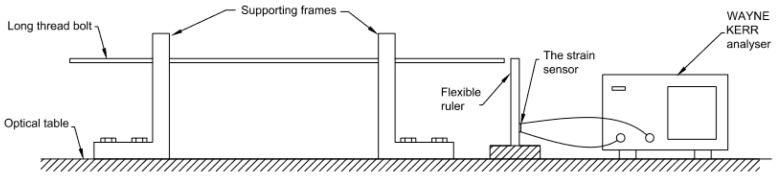
The experiment set up for cantilever test for the strain sensor.

For the configuration in Figure 8, the sensor is positioned at the right hand side, so the value for *y* is given as:

(17) $$y=\frac{t}{2}$$

where *t* is the thickness of the cantilever. Hence, the strain applied to the sensor is:

(18) $$\varepsilon_{z}=-\frac{3D_{y}}{l_{c}^{3}}\cdot \frac{t}{2}\cdot \left(l_{c}-z\right)$$

when the deflection is from the left to the right, $D_{y}$ will be positive, $\varepsilon_{z}$ will be negative and the strain sensor is under a compressed condition, while, when the deflection is from the right to the left, $D_{y}$ will be negative, $\varepsilon_{z}$ will be positive and the strain sensor is under a tensile condition.

### 4.2. Experimental Set up

A flexible ruler that is made from ultra-flexible polyvinyl chloride (PVC) with 2.23 mm thickness (measured by micro-calliper) was selected as the cantilever. Compared with the normal materials that could have been employed for the cantilever, e.g., steel or aluminium, PVC is much easier to bend and has a wider elastic region. PVC is also capable of being bonded with the PDMS of the strain sensor as described below.

**Figure 9:**
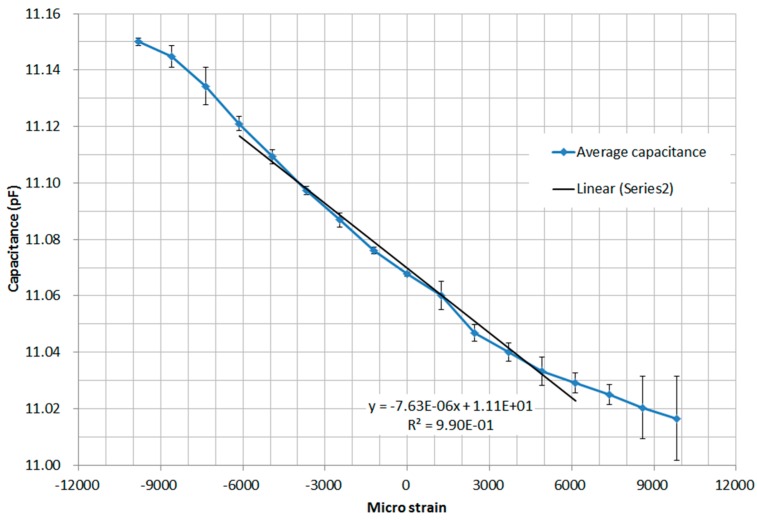
Plot of the average capacitance vs. strain for the cantilever tests reported in Table 1 (error bars based on 3 standard deviation (S.D). are shown).

A long thread bolt that could be easily extended was selected to apply the bending force to the cantilever. Two aluminium frames for supporting the bolt were machined and fixed on an optical table with the bolt threaded through the supports, as shown in Figure 9. One end of the long bolt was pushing the free end of the cantilever and could be adjusted by threading in from the other end. The deflection could be measured directly or was equivalent to the depth of the bolt threading in. The sensor was bonded on to the flexible ruler by a Sil-Poxysilicone adhesive from Smooth-on with primer NO 3 from ACC-silicone, a method applicable to bonding PDMS to many materials including tyre rubber. It was electrically connected by two single wires soldered to the flexible PCB. The connecting wires were fixed either on the ruler or on the table to minimize the influence on the measured capacitance that might be brought by any movements of the wires. The capacitance of the sensor was measured by a WAYNE KERR 6425 Precision Component analyser (Farnell Instruments Limited, Wetherby, UK). The measurement was set as in parallel mode and the measurement speed was set as slow. The frequency of the analyser was set up as 5 kHz and 1 volt alternating voltage was applied on a 2-volt direct voltage. The distance between the fixed and free ends of the cantilever was 10.5 cm and the strain sensor was bonded to the point that was 2 cm away from the fixed end of the cantilever.

### 4.3. Results

The cantilever bending tests were repeated three times to test for repeatability. The results of the experiments are given in Table 1 and the average measured capacitance *versus* applied strain is presented in Figure 9.

The capacitance of the sensor was approximately 11 pF this is higher than capacitance predicted in Section 2.2. This is likely due to parasitic capacitance from the metal tracks on the flexible PCB and the connection wires. It can be seen from the results of the three tests in Table 1, that the measurements were repeatable indicating the sensor was stable with no drift.

There are, however, some limitations for the cantilever test. It can be clearly seen in Figure 9 that the rate of change of capacitance with strain is smaller at strains over ±6,000 micro strain relating to deflections over ~2.5 cm. This is due to the difficulty in applying a force only in the y-direction as the deflection of the end of the cantilever increases, introducing some variability into the measurement. In fact, when the deflection was larger than 4 cm, the long thread bolt could hardly apply a force that was only in y-axis direction due to the over-bended condition of the cantilever. Hence when the deflection is higher than 4mm, the actual strain at the strain sensor is no longer able to be calculated from the Equation (18). Therefore, the cantilever test is not reliable with any larger strain condition that is over ±1% (±10,000 micro strain). To overcome this a thicker cantilever could be used. From Equation (18) it is known that the strain is in a linear relationship with the thickness of the cantilever, so if the thickness of the flexible ruler is doubled, the strain that can be achieved by this method will be approximately ±2%.

The effect of the variability in applying the force is seen as a larger standard deviation at large deflection, e.g., in test 1, the standard deviation at deflection −4 mm is 0.0049 while at deflection −1 mm is only 0.0009. Within ±5000 micro-strain (±½%), the capacitance is seen to have an essentially linear relationship with strain and the standard deviation was approximately 0.001 pF.

Fitting the central part of the capacitance-strain curve as shown in Figure 9 determines a sensitivity for the bond-wire strain sensor of −76 fF per 10,000 micro-strain, which is a better sensitivity than predicted by the theory but which is of the same order of magnitude. The reasons for the difference between the experiment and the theory are likely to be that: (i) the coplanar structure although similar to the capacitive situation of the bond wire array in the sensor but might not describe it exactly; (ii) the actual spacing and height in the wire array might also differ from the original settings; and (iii) the curve of the wires generated by the wire bonding machine might not be a precise arc as assumed for the theoretical calculations.

If the capacitance of the sensor can be measured to a resolution of 1fF *i.e.*, to the standard deviation seen in the cantilever test measurement, the resolution of strain that can be measured will be ~132 micro-strain. Through a separate bending test where an identical sensor (the original sensor is bonded to the cantilever and so is not possible to be used for additional testing) was bent around different diameter rods, it was found that the sensor was capable of measuring strains up to at least 60,000 micro-strain (6%). However, the bending test is not suitable for the strains less than 1% and has higher errors in the measurement, and so does not give an accurate result over the full strain range of interest.

## 5. Experiments on the Strain Sensor in a Vehicle Tyre

**Figure 10:**
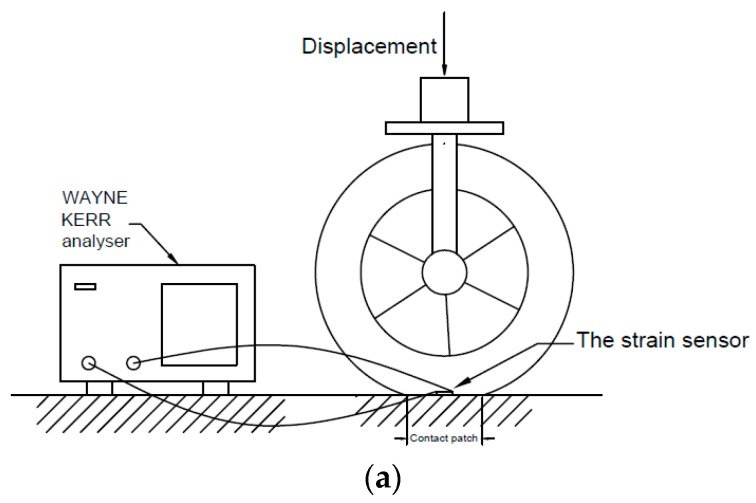

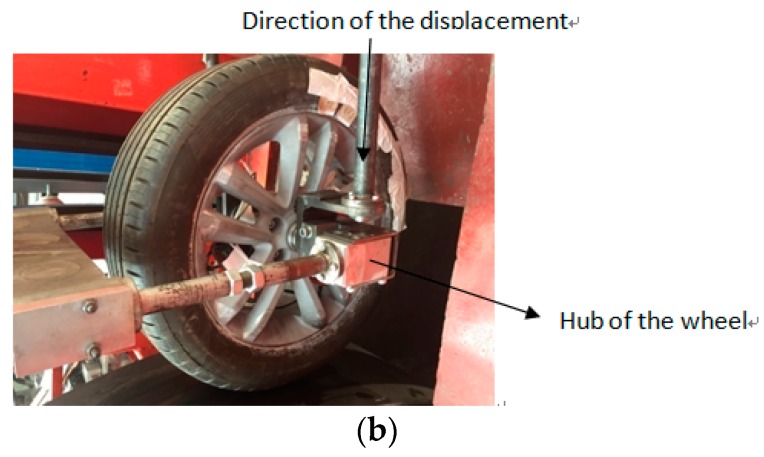
The experimental set-up for tyre test for the strain sensor: (**a**) the schematic diagram; and (**b**) the tyre mounted in the test rig.

To further show the functionality of the sensor an intelligent tyre prototype testing system and a 255/55R, 17 vehicle tyres were employed to test the capacitance changes of the bond wire strain sensor at different loading conditions in a vehicle tyre. The experimental set-up is shown in Figure 10. The strain sensor, which aimed at measuring the tyre strain in the longitudinal direction, was attached on the centre line of the tread in the inner surface of the tyre by the same adhesive method that was used in the last section to attach the sensor to the PVC ruler. Different from traditional adhesive method (e.g., vulcanizing fluid) used in tyre components, there is no need to sand the tyre surface before applying the adhesive. The tyre was then positioned on the tyre test rig such that the sensor was located at the midpoint of the contact patch. The WAYNE KERR 6425 Precision Component analyser was employed to measure the capacitance of the strain sensor with the same settings in the calibration process as mentioned previously. The inflation of the tyre was 32 psi (approximately 2 bar). The displacement was applied to the hub of the wheel as shown in Figure 10 and was varied from zero to 15mm, with more than 2 kN vertical load. The measured capacitance changes are shown below in Table 2.

**Figure 11:**
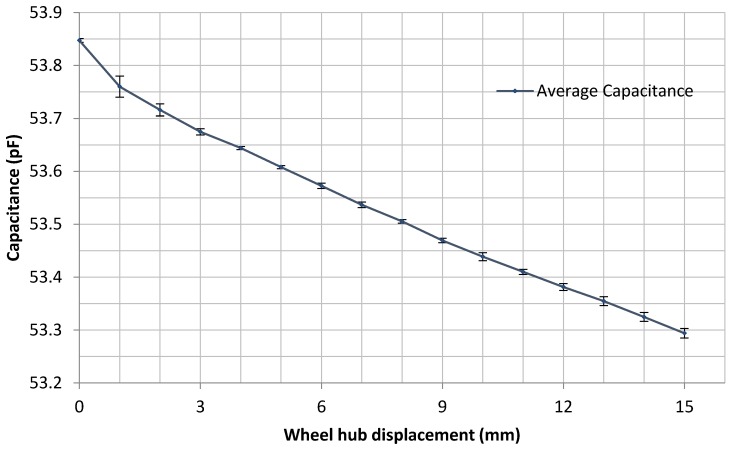
Plot of the average capacitance vs. displacement for the tests reported in Table 2 (error bars based on 3 S.D. are shown).

The initial capacitance was approximately 53.85 pF, which is 40 pF higher than that in the cantilever calibration process. The reason is inferred that the wire connections between the strain sensor and the external measuring devices dramatically increased the parasitic capacitance. However, the capacitance changes were stable along with the increases of the displacements, which can be seen from Figure 11. The measurements were also repeatable, which proves the stability of the sensor when attached inside the tyres.

**Figure 12:**
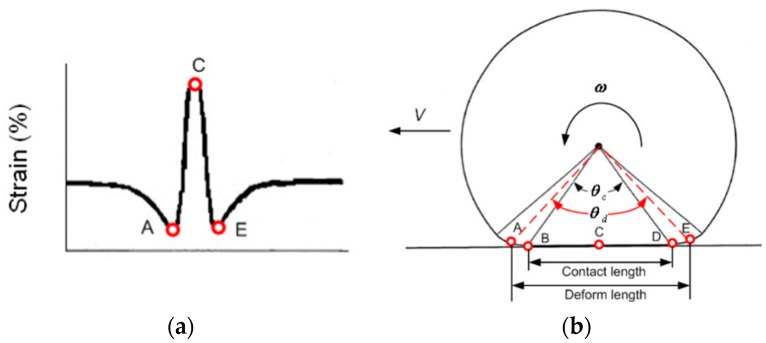
Diagrams showing: (**a**) The tyre longitudinal strain distribution in the contact patch; and (**b**) the side view of the tyre [6].

What is also noticed from the measurement data is that the total capacitance change in the tyre measurement is approximately 0.55 pF, which is much higher than that seen in the cantilever test calibration (0.14 pF). This is due to the large strain at the measuring point in the loaded tyre. The strain distribution along a tyre contact patch is diagrammatically shown in Figure 12. As mentioned in the previous paragraph, the sensor was attached at the midpoint of the contact patch which presents the highest strain position. Results of very initial FEA simulation of the tyre contact patch, using Abaqus 6.13 (not shown here), have indicated that the longitudinal strain in the contact patch under static load are significantly higher than those under dynamic load, and these in dynamic load have been seen to reach up to 3% as confirmed by Lee *et al.* [25]. Hence, it is reasonable to suggest that the higher capacitance changes seen in the in-tyre tests are due to the significant increase in applied strain as compared to the cantilever test. From the results in the tyre test and the calibration test, the strain of the measuring point changes from zero to approximately 8% when displacements are applied from zero to 15 mm.

**Figure 13:**
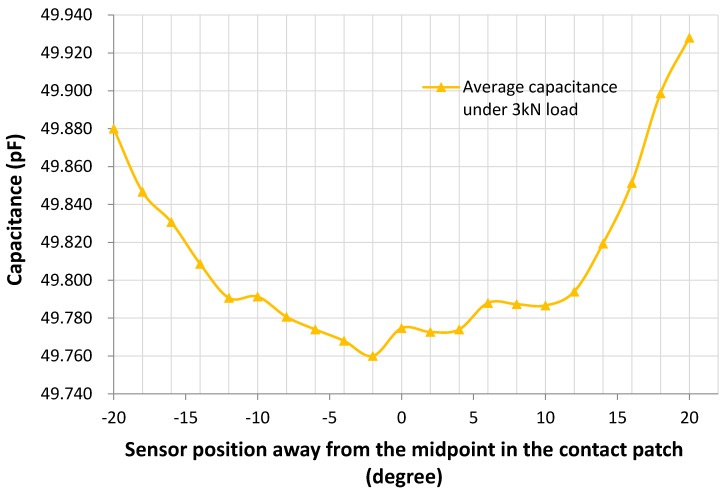
Plot of average capacitance at different positions along the contact patch under 3 kN load for the tests reported in Table 3.

In order to further investigate the capacitance signal along the contact patch, the sensor was positioned and measured at different points along the contact patch in a static condition, by rotating the tyre in two-degree increments away from when the sensor was positioned at the midpoint of the contact patch. The load applied to the tyre was fixed at 3 kN. Three measurements were taken at each rotation angle and the results are shown in Table 3 and plotted in Figure 13.

As shown in Figure 13, the capacitance of the sensor decreased before approaching the midpoint of the contact patch, reached the minimum value at the midpoint and increased after leaving the midpoint of the contact patch. This is as expected as the strain increases toward the centre of the contact patch thus stretching the sensor and reducing the capacitance. This shows that the sensor can successfully detect the strain behaviour along the contact patch and accords with the strain signal in Figure 12. However, the compressed strains shown in Figure 12 when the tyre is entering/leaving the contact patch were not detected by the sensor. This is due to the drum system employed in the test rig. The tyre was compressed to a drum that acts as the road, which means that the strain behaviour along contact patch would be slightly different from the situation that is shown in Figure 12 for a rotating tyre.

Although the sensor shows the general trend of the strain in the contact patch there is still some uncertainty in the measurement data due to the limits of the test rig, and the measurement setup. The connection of the sensor to the component analyser outside the tyre requires wiring inside the tyre, which is then passed through a pressure sealed feedthrough before continuing to the analyser. Movement of this wiring can result in small fluctuations of the measured capacitance. Taping these wires in place can efficiently reduce these errors but it is difficult to eliminate them completely. In addition, due to the limits of the test rig, the load on the tyre must be released and re-loaded at each angular position; hence this has the combined effect of uncertainty in the exact loading as well as potential movement of the wires during this process. The initial capacitance at each rotational position was estimated to vary by up to 0.010 pF. The next phase of our work will aim to develop a wireless measurement and readout system that will look to reduce some of the potential capacitance errors associated with wiring.

## 6. Conclusions

This paper presented a novel flexible strain sensor based on bond wire technology with a measuring range up to at least ±6% and 132 micro-strain (0.013%) resolution shown over a range of ±1% for 1 fF capacitance measurement resolution. Using a cantilever test the sensitivity of the sensor was found to be −76 fF per 10,000 micro-strain. This sensitivity is limited by the spacing of the wires in the array and finer pitch arrays or a higher dielectric constant polymer could improve the sensitivity to strain. As well as cantilever testing, the sensor was also attached on the inside surface of a vehicle tyre and stable results from different displacement conditions were observed, which also proved the repeatability of measuring high strains. The sensor was fabricated using a laser machining process, a wet etching process and a wire bonding process; and finally embedding in flexible PDMS. The flexible nature of the PDMS used in the sensor allows the measurement of strain in tyres without any influence and disturbance to the sensing area. The fabrication processes used are commercial type processes and achieved dimensions in the design with good stability and repeatability, which gives a certain possibility for introducing the sensor into mass production. Tyre sensors are becoming an important part of the intelligent car and recent advances in the form of the Pirelli Cyber Tyre™ show that there is a need for in tyre sensors and that the electronics required for communicating with the car can be applied directly to the tyre. Historically capacitive based in-tyre strain sensors have had limited applicability to intelligent tyres, as operational rotational speeds require high readout rates for positional resolution; and measuring capacitive sensors at high readout speeds whilst maintaining high resolution has not been possible in the tyre. However, advances in commercially available capacitive measurement chips such as the PICOCAP^®^ by ACAM have made the use of such sensors a real prospect. These chips use comparison of discharge rates between a reference capacitor and the sensor capacitor to measure the sensor capacitance at high resolution and high speed; being capable of sub femto-Farad resolution at the required 3 kHz measurement readout speed. The presented bond wire capacitor sensor can be directly connected to such a measurement chip and so integration of the measurement chip on the flexible PCB of the sensor puts a fully integrated in-tyre strain measurement system within our grasp.

## Figures and Tables

**Table 1:** Results of the cantilever test for the strain sensor, showing calculated strain and measured capacitance.

		Capacitance (pF)				
Deflection, $D_{y}$ (cm)	Micro-Strain,$\varepsilon_{z}$	Test 1	Test 2	Test 3	Average	Standard Deviation
4	−9824.43	11.1505	11.1495	11.1500	11.1500	0.0004
3.5	−8596.37	11.1455	11.1460	11.1430	11.1448	0.0013
3	−7368.32	11.1325	11.1375	11.1330	11.1343	0.0022
2.5	−6140.27	11.1200	11.1210	11.1220	11.1210	0.0008
2	−4912.21	11.1090	11.1085	11.1105	11.1093	0.0008
1.5	−3684.16	11.0970	11.0970	11.0980	11.0973	0.0005
1	−2456.11	11.0865	11.0860	11.0880	11.0868	0.0008
0.5	−1228.05	11.0760	11.0755	11.0765	11.0760	0.0004
0	0	11.0680	11.0675	11.0675	11.0677	0.0002
−0.5	1228.05	11.0580	11.0605	11.0620	11.0602	0.0016
−1	2456.11	11.0475	11.0475	11.0455	11.0468	0.0009
−1.5	3684.16	11.0390	11.0415	11.0395	11.0400	0.0011
−2	4912.21	11.0310	11.0345	11.0345	11.0333	0.0016
−2.5	6140.27	11.0275	11.0300	11.0300	11.0292	0.0012
−3	7368.32	11.0235	11.0265	11.0250	11.0250	0.0012
−3.5	8596.37	11.0160	11.0250	11.0200	11.0203	0.0037
−4	9824.43	11.0095	11.0200	11.0200	11.0165	0.0049

**Table 2:** Results of the capacitance changes of the strain sensor with respect to different displacements.

	Capacitance (pF)				
Displacement (mm)	Test 1	Test 2	Test 3	Average	Standard Deviation
0	53.846	53.852	53.844	53.8473	0.0034
1	53.732	53.776	53.772	53.7600	0.0199
2	53.700	53.724	53.724	53.7160	0.0113
3	53.668	53.674	53.682	53.6747	0.0057
4	53.642	53.648	53.642	53.6440	0.0028
5	53.610	53.610	53.604	53.6080	0.0028
6	53.578	53.574	53.566	53.5727	0.0050
7	53.544	53.532	53.534	53.5367	0.0052
8	53.510	53.502	53.504	53.5053	0.0034
9	53.474	53.464	53.470	53.4693	0.0041
10	53.446	53.428	53.442	53.4387	0.0077
11	53.416	53.404	53.410	53.4100	0.0049
12	53.390	53.374	53.380	53.3813	0.0066
13	53.366	53.346	53.352	53.3547	0.0084
14	53.336	53.316	53.322	53.3247	0.0084
15	53.306	53.284	53.292	53.2940	0.0091

**Table 3:** Results of capacitances of the strain sensor along the contact patch under 3 kN load.

	Capacitance (pF)				
Sensor Position Away from the Midpoint (Degree)	Test1	Test 2	Test 3	Average	Standard Deviation
20	49.872	49.92	49.992	49.928	0.0493
18	49.826	49.91	49.96	49.899	0.0553
16	49.748	49.872	49.934	49.851	0.0773
14	49.708	49.846	49.904	49.819	0.0822
12	49.682	49.814	49.886	49.794	0.0845
10	49.68	49.794	49.886	49.787	0.0843
8	49.69	49.788	49.884	49.787	0.0792
6	49.7	49.784	49.88	49.788	0.0735
4	49.682	49.772	49.868	49.774	0.0759
2	49.68	49.774	49.864	49.773	0.0751
0	49.676	49.786	49.862	49.775	0.0764
−2	49.64	49.77	49.87	49.760	0.0942
−4	49.644	49.78	49.88	49.768	0.0967
−6	49.648	49.782	49.892	49.774	0.0998
−8	49.656	49.786	49.9	49.781	0.0997
−10	49.662	49.8	49.912	49.791	0.1022
−12	49.68	49.792	49.9	49.791	0.0898
−14	49.7	49.81	49.916	49.809	0.0882
−16	49.736	49.836	49.92	49.831	0.0752
−18	49.75	49.854	49.936	49.847	0.0761
−20	49.816	49.87	49.954	49.880	0.0568

## Abbreviations

The following abbreviations are used in this manuscript:

FEA	Finite Element Analysis
PVDF	Polyvinylidene fluoride
PSD	Position Sensitive Detector
PDMS	Polydimethysiloxane
PCB	Printed Circuit Board
PVC	Polyvinyl Chloride
